# The value of the 2011 ASAS classification criteria in patients with Spondyloarthritis and the prognosis of non-radiographic axial Spondyloarthritis: data from a large cohort of a tertiary referral hospital

**DOI:** 10.31138/mjr.30.1.51

**Published:** 2019-03-28

**Authors:** Nikolaos Kougkas, Nestor Avgoustidis, Argyro Repa, George Bertsias, Anastasios Eskitzis, Prodromos Sidiropoulos

**Affiliations:** Department of Rheumatology, Clinical Immunology and Allergy, University of Crete School of Medicine, Heraklion, Greece

**Keywords:** Spondyloarthritis, classification criteria, non-radiographic, prognosis

## Abstract

Spondyloarthritides (SpA) are a group of interrelated rheumatic disorders that includes ankylosing spondylitis (AS), psoriatic arthritis (PsA), arthritis related to inflammatory bowel disease and reactive arthritis. Since the latest classification criteria published from the ASAS (Assessment of SpondyloArthritis international Society), patients with these diagnoses can be classified either as having axial or peripheral SpA. In this study, these new criteria of ASAS will be applied to all patients with a clinical diagnosis of SpA that are followed in the Rheumatology Clinic of University Hospital of Heraklion. Furthermore, patients with non-radiographic axial SpA (nrAxSpA) will be monitored, both retrospectively and prospectively, for their long-term outcome in terms of imaging and clinical aspects (remission, disability, severe complications, eg, uveitis). This study is expected to give valuable information of the performance of these new criteria in daily clinical practice and of the prognosis of patients with non-radiographic axial SpA.

## INTRODUCTION

Spondyloarthritides consist of a heterogeneous group of inflammatory diseases that share common clinical, radiological and genetic features. During the last decades a tremendous progress has been achieved in the field of diagnostic approach of spondyloarthropathies by the application of magnetic resonance imaging (MRI), as well as in further understanding their pathogenesis basis (genetic background, inflammatory pathways and novel cytokines).

As a result of this progress, there have been important changes in the diagnosis, classification and treatment of these diseases.

In 2009 and later on in 2011, the ASAS published a new set of classification criteria for spondyloarthropathies according to clinical features and imaging that classify them in to two groups, axial and peripheral SpA.^1,2^ While axial SpA mainly corresponds to the “classic” term “Ankylosing Spondylitis” which is the prototype of SpA, the characterization of peripheral SpA was a major step, since it comprises most of the cases diagnosed in the past as “undifferentiated SpA”. This is considered clinically important because it allows for an earlier and more accurate diagnosis of patients with clinical features of SpA. Moreover, by applying the MRI in patients with chronic low back pain and normal X rays, a new entity (the “non-radiographic axial Spondyloarthritis” [nrAxSpA]) has well been recognized.^3^ Even today, it is not yet clear whether the latter form represents a distinct disease or if it represents an early stage of Ankylosing Spondylitis.^4^

## AIM OF THE STUDY

In the present study we aim to:

1. Assess the value of the 2011 ASAS classification criteria in clinical practice, focusing in patients with a former diagnosis of undifferentiated SpA. For that aim we will apply the new classification criteria retrospectively and prospectively in a large cohort (n=600) of patients with spondyloarthropathies.
2. To establish a registry of patients with nrAxSpA with a detailed follow-up in order to assess the long-term outcome regarding treatment, quality of life and the transition rate to the radiographic form of the disease (AS).

## METHODS

The study will take place in the Rheumatology Clinic of the University Hospital of Heraklion. All patients with a diagnosis of SpA who are followed in our department (including those with undifferentiated SpA, PsA, enteropathic and reactive arthritis) will be analyzed. All patients’ files will be reviewed, and data will be entered in the Registry of the Clinic (Rheumatology Clinic Data Base - RCDB) in order to be analyzed. Diagnosis and time to classification according to the latest ASAS criteria either as Axial or as Peripheral SpA will be recorded. We will assess:

1. The value of the new criteria to re-classify patients with a diagnosis of undifferentiated SpA.
2. Their baseline characteristics, treatments and outcome.

Re-classified patients as peripheral SpA will be compared to those not fulfilling ASAS criteria.

Patients with nrAxSpA will be assessed for:

1. Possible transition to radiographic SpA based on plain X-rays.
2. The use of biologic treatment compared to those patients with AS.
3. Outcome, concerning disease activity, quality of life and functional status.

This part of the study will have a retrospective and a prospective arm. All patients will have a minimum of 2 years follow-up in order to better characterize their outcome.

Demographics, treatments, disease’s characteristics (BASDAI, ASDAS, DAPSA) function (BASFI, HAQ) and quality of life (EUROQUOL 5D) will be retrieved or prospectively documented accordingly.

We estimate to record approximately 600 existing and 50 new SpA patients per year.

## IMPORTANCE OF THE STUDY

There are not many data assessing the application in clinical practice of the aforementioned ASAS new proposed classification criteria. Moreover, there are limited data about SpA as a group of diseases in the Hellenic population, and generally in the Mediterranean area. A recent study from the Hellenic Registry for Biologic Therapies (HeRBT) has shown the 10-years response rate and outcome of Tumor Necrosis Factor inhibitors (TNFi) in patients with SpA.^5^ The importance of the current study consists in assessing the usefulness in everyday clinical practice of the new ASAS criteria to classify patients with SpA. We will aim to assess whether ASAS criteria have any additional benefit compared to the earlier criteria in classifying patients with undifferentiated SpA. Moreover, we will assess the characteristics and the outcome of patients classified as nrAxSpa, and we will evaluate whether early recognition of patients may result in better prognosis.

## STUDY APPROVAL

The Ethics Committee of the University Hospital of Iraklion has approved the study.
